# Inactivated *Pseudomonas aeruginosa* inhibits hypoxia-induced pulmonary hypertension by preventing TGF-β1/Smad signaling

**DOI:** 10.1590/1414-431X20165526

**Published:** 2016-08-25

**Authors:** S.D. Chai, T. Liu, M.F. Dong, Z.K. Li, P.Z. Tang, J.T. Wang, S.J. Ma

**Affiliations:** Department of Cardiac Surgery, Liaocheng People's Hospital, Clinical School of Taishan Medical University, Liaocheng, Shandong Province, China

**Keywords:** Inactivated *Pseudomonas aeruginosa*, Pulmonary hypertension, TGF-β/Smad, Primary arterial smooth muscle cells, α-smooth muscle actin

## Abstract

*Pseudomonas aeruginosa* is one of the common colonizing bacteria of the human body and is an opportunistic pathogen frequently associated with respiratory infections. Inactivated *P. aeruginosa* (IPA) have a variety of biological effects against inflammation and allergy. Transforming growth factor-β (TGF-β) signaling plays a critical role in the regulation of cell growth, differentiation, and development in a wide range of biological systems. The present study was designed to investigate the effects of IPA on TGF-β/Smad signaling *in vivo,* using a hypoxia-induced pulmonary hypertension (PH) rat model. Sprague Dawley rats (n=40) were exposed to 10% oxygen for 21 days to induce PH. At the same time, IPA was administered intravenously from day 1 to day 14. Mean pulmonary artery pressure (mPAP) and the right ventricle (RV) to left ventricle plus the interventricular septum (LV+S) mass ratio were used to evaluate the development of PH. Vessel thickness and density were measured using immunohistochemistry. Primary arterial smooth muscle cells (PASMCs) were isolated and the proliferation of PASMCs was assayed by flow cytometry. The production of TGF-β1 in cultured supernatant of PASMCs was assayed by ELISA. The expression levels of α-smooth muscle actin (α-SMA), TGF-β1 and phospho-Smad 2/3 in PASMCs were assayed by western blot. Our data indicated that IPA attenuated PH, RV hypertrophy and pulmonary vascular remodeling in rats, which was probably mediated by restraining the hypoxia-induced overactive TGF-β1/Smad signaling. In conclusion, IPA is a promising protective treatment in PH due to the inhibiting effects on TGF-β1/Smad 2/3 signaling.

## Introduction

Pulmonary hypertension (PH) is a disease characterized by increased cell proliferation and remodeling of the vascular wall leading to increased pulmonary artery pressure that results in right ventricle hypertrophy and subsequent heart failure (1). Imbalance between vasoconstrictors and vasodilators induced by vascular dysfunction has been shown to play crucial roles in the pathogenesis of PH, in which the endogenous production of pulmonary vasodilators such as endothelial nitric oxide synthase, prostanoids etc. decrease while vasoconstrictors such as endothelin-1 (ET-1), thromboxane and serotonin etc. increase (2). In addition to this, multiple other signaling pathways are confirmed to be associated with pulmonary hypertension including increased apelin and fibroblast growth factor 2 in pulmonary artery endothelial cells (3), endothelium-specific tyrosine kinase-2 receptor activation by angiopoietin-1 (4), activation of Notch (5) etc.

Vasodilators including ET-1 receptor antagonists, prostacyclin analogs, phosphodiesterase type 5 inhibitors, and rho-kinase inhibitors are common agents utilized to decrease pulmonary vascular resistance and to relieve PH. Furthermore, right ventricle-targeted therapies, which aim at mitigating the effects of functional right ventricular failure, include β-adrenoceptor blockers, angiotensin-converting enzyme inhibitors, antioxidants, modulators of metabolism, and 5-hydroxytryptamine-2B receptor antagonists (6,7). However, the therapeutic effects remain challenging because the symptoms of PH usually become pronounced only in the late stages of the disease.

*P. aeruginosa* is one of the common colonizing bacteria of the human body, which is frequently present in chronic infection of the respiratory tract. Based on animal experiments, using TLR knockout mice, *P. aeruginosa* is believed to recognize TLR-2, -4, and -5 on epithelial cells through its lipopolysaccharide and flagellin (8,9). Also, recent reports showed that inactivated *P. aeruginosa* (IPA) can decrease airway inflammation, improve epithelial functions and stimulate recovery from abnormal airway microenvironment (10). Moreover, IPA inhibits the secretion of nodal, a type of secreting protein belonging to the transforming growth factor-β (TGF-β) superfamily, which is tightly regulated by various microbes in lungs and promotes proliferation and epithelial-mesenchymal transition of epithelial cells through DNA methylation (11).

TGF-β1 is a multifunctional cytokine, a potent inhibitor of epithelial cell repair and an inducer of a hypertrophic and hypercontractile arterial smooth muscle cells (ASMCs) phenotype through regulating cell proliferation, growth, differentiation, cells movement, immunomodulatory effects and profibrogenic effects (12). The classical Smads pathways (receptor-mediated Smads, Smad 2/3), mitogen-activated protein kinase as well as nuclear factor kappa B signaling, have been reported to be involved in the modulation of ASMCs proliferation and migration, induced by TGF-β1 (13,14). Recent studies have shown that exogenously applied TGF-β1 alone promoted a contractile ASMC phenotype through the activation of Smad signaling (15).

According to the above information, the present study was designed to observe the effects of IPA on proliferation of PASMCs and corresponding TGF-β1 signaling *in vivo* and *in vitro*.

## Material and Methods

### Animals and experimental design

All animal experiments and procedures were approved by the Institutional Animal Care and Use Committees of Liaocheng People's Hospital. Sprague Dawley rats (n=40, male, age 5–6 weeks) were purchased from Liaocheng People's Hospital. IPA was obtained from Beijing Wanteer Biological Pharmaceutical Co., Ltd., China, which is mainly used in clinical treatments of chronic bacterial infections. Animals were divided into four groups of 8–10 animals, including normal control group, hypoxia group, control+IPA group and hypoxia+IPA group. The hypoxia groups were exposed to 10% O_2_ and the control groups to room air, all under a natural light cycle for 21 consecutive days. Animals in the IPA-treated groups were intravenously injected with 200 μL of IPA (1×10^7^ cfu/mL) for 14 consecutive days, while animals without IPA treatment were intravenously injected 0.2 mL saline for 14 consecutive days.

### Measurement of PH

Twenty-one days after hypoxic exposure, the rats were weighed and anesthetized with 3% pentobarbital sodium (60 mg/kg body weight) by intraperitoneal injection. A polyethylene catheter with a diameter of 1 mm was gradually inserted into the pulmonary artery through an incision in the right external jugular vein and the mean pulmonary artery pressure (mPAP) was recorded by a hemodynamic analyzing system (RM-6200C, Chengdu, China). The rats were then anatomized and the left ventricle plus the interventricular septum (LV+S) and the right ventricle (RV) tissue were collected by cutting along the edge of the ventricle and the interventricular septum, and weighed. The RV/(LV+S) mass ratio was used as the index for RV hypertrophy.

### Immunohistochemistry for tissue morphology

Lungs were inflated, harvested, fixed in 2% paraformaldehyde, and embedded in paraffin. To visualize the pulmonary medial arterial wall of pulmonary vessels, lung sections were immunostained with a polyclonal rabbit anti-α-smooth muscle actin (α-SMA) antibody at a dilution of 1:200 (Abcam, USA) for 3 h at 4°C. To quantify the density of pulmonary vessels, a polyclonal rabbit anti-von Willebrand factor (vWF) antibody (Santa Cruz Biotechnology, USA) was employed as an endothelial marker at a dilution of 1:400. The slides were washed and incubated with biotinylated goat anti-rabbit IgG for 1 h and washed again. After washing in PBS, the signal was detected with 3,3'-diaminobenzidine (Dingguo, China). α-SMA-stained tissue sections were captured at ×400 magnification and the thickness of the vessel wall was calculated using the following equation: ((area1−area2)/area1)×100, where area1 and area2 are the areas within the external and internal boundaries of the α-SMA layer, respectively. To quantify vessel density, the number of vWF-positive vessels per high-power field was counted at a magnification of ×100.

### Cell isolation and culture

To test directly the effects of IPA on TGF-β1 under hypoxia, isolated primary arterial smooth muscle cells (PASMCs) were used. PASMCs were isolated from distal segments of rat pulmonary arteries (0.1–0.2 mm external diameter) using the method described previously (16). PASMCs were used for experiments at passages 3 to 4. Before each study, PASMCs were subjected to serum starvation for 24 h. To examine the effects of hypoxia on TGF-β1 protein expression, PASMCs were transferred into an air-tight hypoxic incubator containing 5% CO_2_ and 1% oxygen for 24 h.

### ELISA assay

The level of TGF-β1 in supernatant of cultured rat PASMCs was measured by ELISA according to the manufacturer's instructions. Briefly, 100 μL supernatant was added to a 96-well microplate coated with capture antibody overnight at room temperature, followed by 100 μL enzyme-linked antibodies incubation for 30 min at 37°C. After three washes, the substrate solution was added and incubated for 20 min. After the addition of stop solution, the absorbance of each well was determined immediately with the use of a microplate reader set to 450 nm. Each sample was repeated three times.

### Western immunoblotting

For western blotting, protein concentrations of cell lysates were determined with a Coomassie plus protein assay (Dingguo) and were processed by 10% SDS-polyacrylamide gel electrophoresis and then transferred onto polyvinylidene fluoride membrane (Invitrogen, China). Membranes were blocked with 5% skim milk and incubated with goat anti-TGF-β1 (Santa Cruz Biotechnology), goat anti-phospho-Smad 2/3 (Santa Cruz Biotechnology) and rabbit anti-α-SMA (Abcam). After being washed, membranes were incubated with corresponding horseradish peroxidase-conjugated IgG (1:5,000; Santa Cruz Biotechnology) for 1 h at room temperature. Antibody-antigen complexes were then detected using an ECL chemiluminescent detection system (Gene Co., Ltd., China). GAPDH was used as a loading control. A densitometry analysis was performed using AlphaEase software version 2200 (Alpha Innotech Co., USA).

### Measurement of cell cycle by flow cytometry

After various treatments, PASMCs were fixed in cold 70% ethanol and stored at -20°C overnight. The fixed cells were washed twice with PBS, stained in a propidium iodide solution (50 µg/mL, Sigma, USA) for 1 h, and treated with a ribonuclease solution (20 µg/mL, Sigma) for 30 min. Flow cytometry (BD Pharmingen, USA) was then used to examine cell cycles.

### Statistical analysis

Data are reported as means±SD. Between-group mean comparisons were performed using one-way analysis of variance (ANOVA). A P-value less than 0.05 was considered to be statistically significant.

## Results

**Figure 1:**
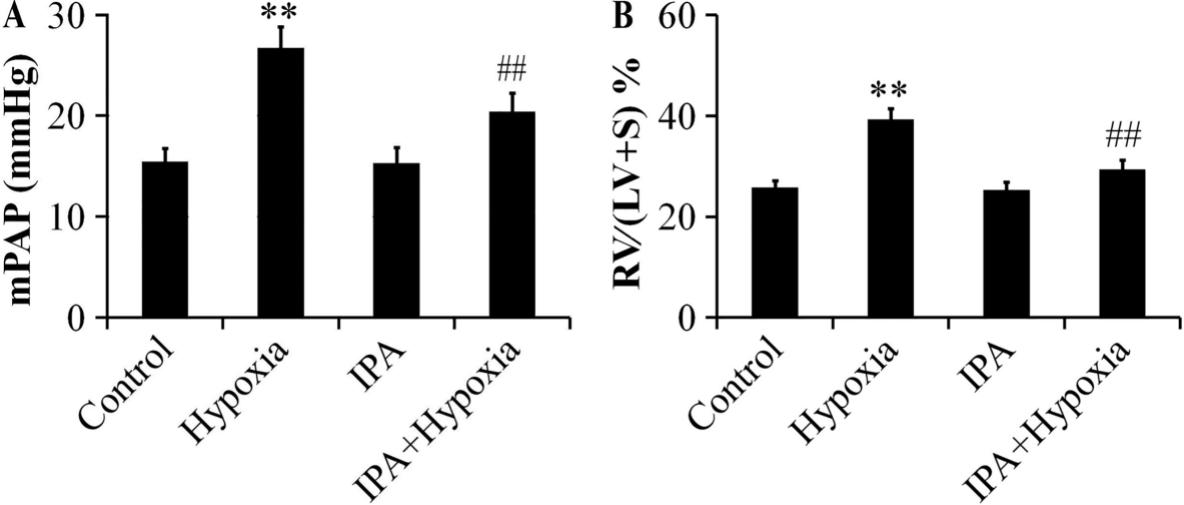
Effects of inactivated *P. aeruginosa* on mean pulmonary artery pressure (mPAP) (*A*) and on the right ventricle (RV)/left ventricle plus the interventricular septum (LV+S) thickness ratio (*B*) in rats (n=6). Hypoxia induced an increase of mPAP (P<0.01), and treatment with IPA effectively attenuated this increase. The RV/(LV+S) % was markedly elevated in the rats in hypoxia group and IPA attenuated the RV/(LV+S) %. Data are reported as mean ± SD. **P<0.01 vs control; ##P<0.01 vs hypoxia (ANOVA).

We measured mPAP and thickness ratio to reflect the hemodynamic changes in rats with induced PH. mPAP increased from 15.99±2.58 to 25.97±1.33 mmHg following the induction of chronic hypoxia (P<0.01), but treatment with IPA effectively decreased mPAP to 18.32±2.47 mmHg (P<0.01; Figure 1A). The RV/(LV+S) % was markedly elevated in the rats in the hypoxia group (39.33±1.34%) compared with the rats in the control group (25.78±2.02%) (P<0.01). Following the injection of IPA, the RV/(LV+S) % decreased to 29.37±1.89% and right ventricular hypertrophy was significantly attenuated (P<0.01; Figure 1B).

**Figure 2:**
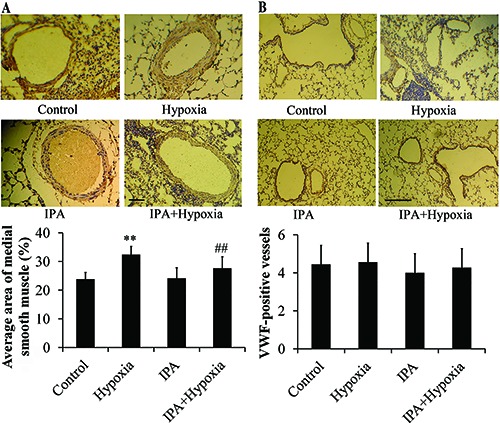
Effects of inactivated *P. aeruginosa* (IPA) on vessel thickness (*A*) and density (*B*), assayed by immunohistochemistry (n=6). The thickness of the arteries in chronic hypoxia rats was significantly increased compared to control rats. IPA markedly attenuated this effect (magnification ×400). **P<0.01 *vs* control; ##P<0.01 *vs* hypoxia (ANOVA). The density of blood vessels, addressed by immunostaining of the von Willebrand factor (vWF), was not different between groups (magnification ×100).

To explore whether hypoxia would influence endothelial and smooth muscle cell development, we analyzed the expression of α-SMA and vWF. The results of immunohistochemistry using the α-MSA marker showed that artery thickness of rats that underwent chronic hypoxia was much more pronounced than that of the control rats. IPA markedly attenuated artery thickness (P<0.01; Figure 2A). IPA had no influence on the artery thickness of the normal group. vWF is normally expressed in the endothelial cells of larger blood vessels within the lung. Our results of vWF immunostaining showed that the density of blood vessels were not different between groups (Figure 2B).

### IPA inhibited proliferation of PASMCs

**Figure 3:**
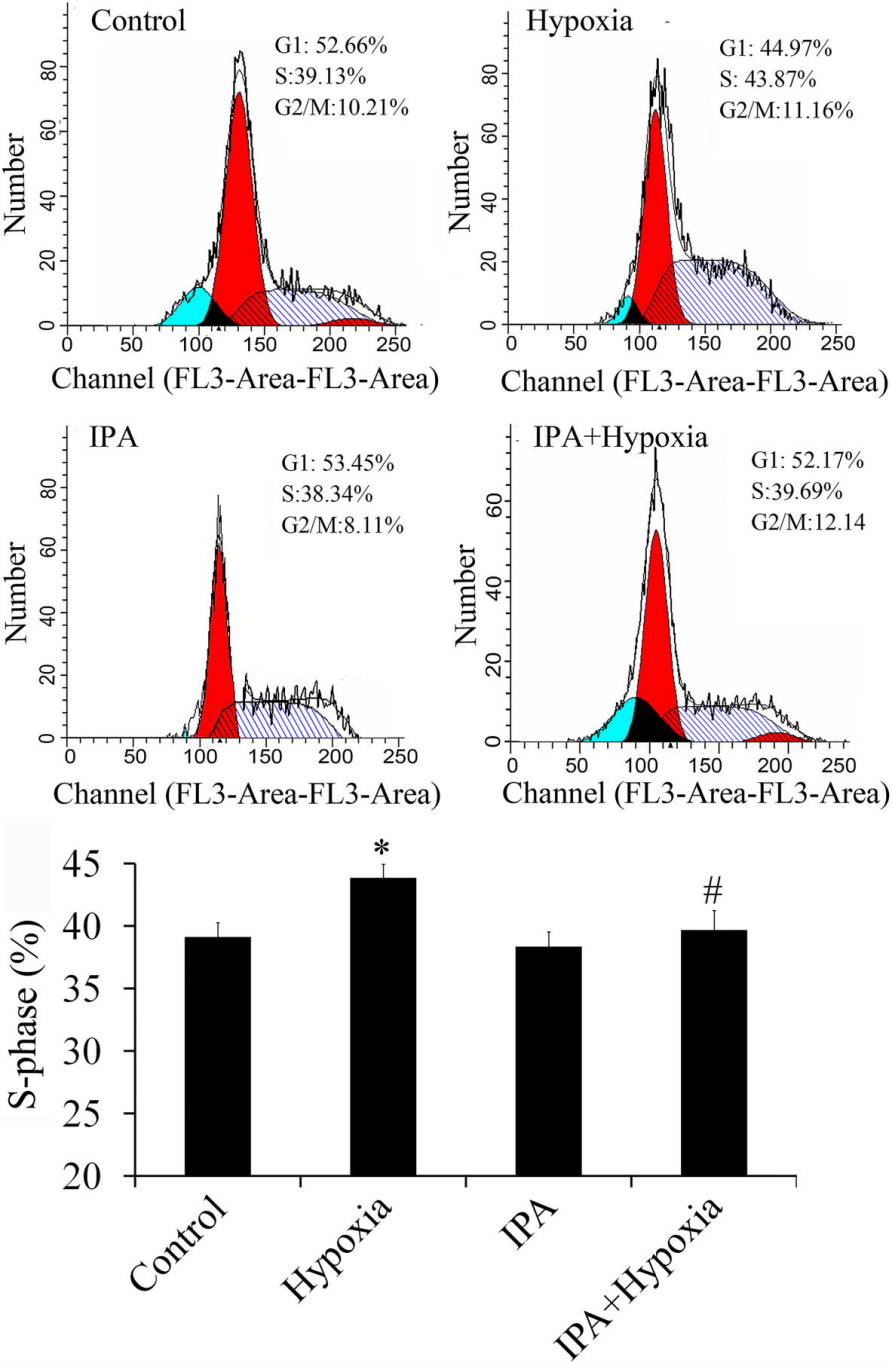
Effects of inactivated P. aeruginosa (IPA) on proliferation of primary arterial smooth muscle cells (PASMCs) (n=4) in rats. Hypoxia promoted the proliferation of PASMCs, and IPA inhibited the proliferation of PASMCs induced by hypoxia. *P<0.05 vs control; #P<0.05 vs hypoxia (ANOVA).

Hypertrophic and hypercontractile arterial smooth muscle cells is one of the main characteristics of PH. Flow cytometry showed that hypoxia promoted the proliferation of PASMCs and IPA had an inhibitory effect (Figure 3).

### IPA inhibited the expression of TGF-β1 and α-SMA

**Figure 4:**
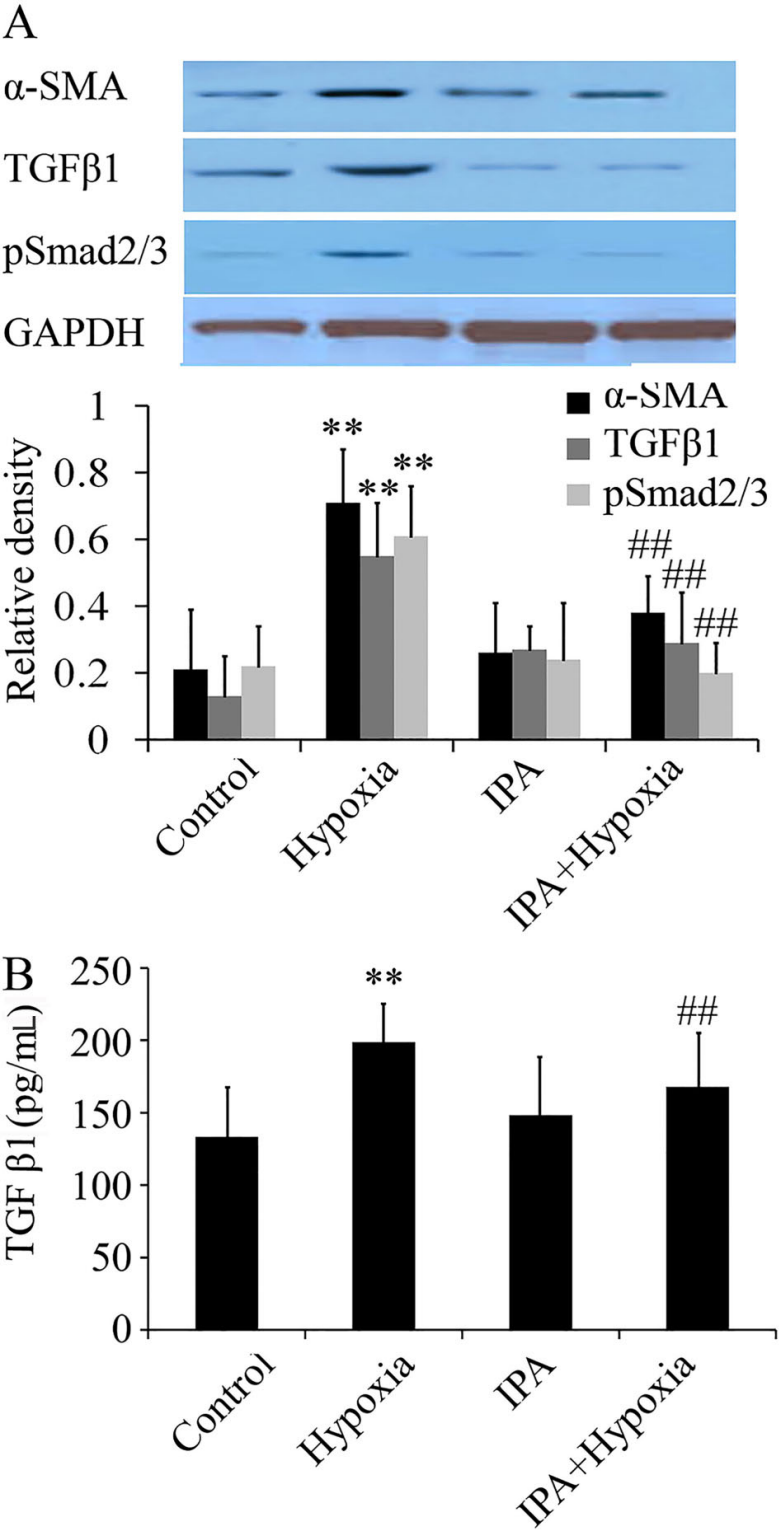
Effects of inactivated *P. aeruginosa* (IPA) on TGF-β1 signaling and α-SMA protein expression, assayed by western blot (*A*) and levels of TGF-β1 in supernatant, assayed by ELISA (*B*) (n=6) in rats. Hypoxia increased TGF-β1/Smad signaling and α-SMA protein expression at 24 h, and IPA inhibited the expression of TGF-β1/Smad signaling and α-SMA induced by hypoxia. Moreover, the production of TGF-β1was up-regulated in the hypoxia group and IPA inhibited the production of TGF-β1. **P<0.01 *vs* control; ##P<0.01 *vs* hypoxia (ANOVA).

Based on previous studies, it has been confirmed that TGF-β1 is the key factor in the proliferation of smooth muscle and remodeling of blood vessels (17). To examine the role of TGF-β1 signaling in IPA-induced protection, we measured protein expression of TGF-β1, and the activation of p-Smad 2/3 and α-SMA in PASMCs exposed to hypoxia (1% O_2_). As shown in Figure 4A, hypoxia increased TGF-β1 signaling and α-SMA protein expression at 24 h, and IPA inhibited both markers. Furthermore, compared with the control group, the production of TGF-β1 in supernatant was up-regulated in the hypoxia group but inhibited in the IPA group (Figure 4B).

## Discussion

Hypoxia plays a key role in the pathogenesis of PH. The pathological changes include pulmonary vasoconstriction, increased proliferation and resistance to apoptosis of smooth muscle cells. Subsequent structural remodeling of the heart and pulmonary vessels has been noted in all types of PH regardless of its causes (18,19). In recent years, accumulating evidence has suggested that the development of hypoxia-induced pulmonary hypertension is associated with vascular remodeling, which is accompanied by the proliferation and migration of both PASMCs and endothelia cells. TGF-β1 is a pro-inflammatory factor and also a growth factor that inhibits the wound repair of bronchial epithelial cells and promotes the vascular remodeling and vasoconstriction. It has been perceived as an important regulator in the pathogenesis of inflammatory pulmonary conditions such as asthma, COPD, PH, etc. (20,21). Elevated levels of TGF-β1 expression and Smad2/3 phosphorylation have been reported in rat models of experimental PH in response to hypoxia, as well as in patients with idiopathic PH (22). On a cellular level, TGF-β1 has been shown to promote the proliferation and migration of PASMCs from rats and patients with PH and to increase extracellular matrix and endothelin-1 expression in rat and human PASMCs (23). In the present study, we found an elevated expression of TGF-β1 and phosphorylation of Smad2/3 in rat lungs after hypoxia exposure, and IPA markedly inhibited the increased expression of TGF-β1 and phosphorylation of Smad2/3, indicating that IPA is potentially a novel and effective inhibitor of hypoxia-induced remodeling.

Moreover, in the present study, we observed that the immunostaining for vWF decreased in chronic hypoxia, and increased with IPA, indicating that IPA promoted endothelial proliferation and wound repair. Endothelial cells accentuate TGF-β1-driven epithelial-to-mesenchymal transition in the airway (24); they may also inhibit proliferation of PASMCs by releasing many kinds of vasodilators (25). However, the specific protective mechanism of IPA still needs to be studied further.

In summary, our results indicate a novel function of IPA in inhibiting smooth muscle cells in the presence of hypoxia. Given the preventive effect to promote endothelial wound repair and inhibit proliferation of ASMCs, we propose that IPA be seen as a promising protective treatment in PH.
